# Are daylight saving time transitions associated with changes in myocardial infarction incidence? Results from the German MONICA/KORA Myocardial Infarction Registry

**DOI:** 10.1186/s12889-015-2124-4

**Published:** 2015-08-14

**Authors:** Inge Kirchberger, Kathrin Wolf, Margit Heier, Bernhard Kuch, Wolfgang von Scheidt, Annette Peters, Christa Meisinger

**Affiliations:** Central Hospital of Augsburg, MONICA/KORA Myocardial Infarction Registry, Stenglinstr. 2, D-86156 Augsburg, Germany; Helmholtz Zentrum München, German Research Center for Environmental Health (GmbH), Institute of Epidemiology II, Ingolstädter Landstr. 1, 85764, Neuherberg, Germany; Hospital of Nördlingen, Department of Internal Medicine/Cardiology, Stoffelsberg 4, 86720 Nördlingen, Germany; Central Hospital of Augsburg, Department of Internal Medicine I – Cardiology, Stenglinstr. 2, D-86156 Augsburg, Germany

## Abstract

**Background:**

Some studies suggest that transitions to and from daylight saving time (DST) have an influence on acute myocardial infarction (AMI) incidence. However, the available publications have a number of limitations e.g. regarding sample size, exclusion of fatal AMI cases, precise assessment of AMI onset, and consideration of possible confounders, and they were conducted in countries with different geographical location. The objective of this study was to examine the association of DST transitions with AMI incidence recorded in the population-based German MONICA/KORA Myocardial Infarction Registry.

**Methods:**

The study sample consisted of 25,499 coronary deaths and non-fatal AMI cases aged 25–74 years. We used Poisson regression with indicator variables for the 3 days or the week after the spring and the autumn transition and adjusted for potential confounders to model the association between DST transitions and AMI incidence. In addition, we built an excess model by calculating observed over expected events per day.

**Results:**

Overall, no significant changes of AMI risk during the first 3 days or 1 week after the transition to and from DST were found. However, subgroup analyses on the spring transition revealed significantly increased risks for men in the first 3 days after transition (RR 1.155, 95 % CI 1.000–1.334) and for persons who took angiotensine converting enzyme (ACE) inhibitors prior to the AMI (3 days: RR 1.489, 95 % CI 1.151–1.927; 1 week: RR 1.297, 95 % CI 1.063–1.582). After the clock shift in autumn, patients with a prior infarction had an increased risk to have a re-infarction (3 days: RR 1.319, 95 % CI 1.029–1.691; 1 week: RR 1.270, 95 % CI 1.048–1.539).

**Conclusions:**

Specific subgroups such as men and persons with a history of AMI or prior treatment with ACE inhibitors, may have a higher risk for AMI during DST. Further studies which include data on chronotype and sleep duration are needed in order to confirm these results.

## Background

More than 1.5 billion people in over 70 countries worldwide are subject of transitions to daylight saving time (DST) since some decades. In Germany, DST was introduced in 1980. It begins on the last Sunday in March. The end was on the last Sunday in September from 1980–1995, and is one month later since 1996.

Very little is known about the effects of the disruption of circadian rhythms caused by DST shifts and the study results are overall inconsistent [1–5]. Studies on DST time shift, however, provide a unique possibility to investigate these effects in a “natural experiment” without confounding from individual characteristics since everyone is exposed at a given time point.

Some published studies have addressed the impact of transitions to DST on acute myocardial infarction (AMI) incidence [6–9]. Data from the Swedish AMI registry showed a significantly higher AMI incidence for the first 3 workdays and the whole week after the spring transition. In contrast, after the autumn transition only the Monday was affected significantly and showed a reduced AMI incidence [6]. However, this study only investigated age and sex as factors that might influence the association between DST time shifts and AMI incidence. A further study from the Swedish authors examined subgroups of AMI cases and found a higher risk of AMI for the spring transition in individuals taking cardiac medication or having low blood lipids, and a lower risk of AMI for the autumn transition in persons with hyperlipidemia, and persons taking statins or calcium-channel blockers, but these differences were not statistically significant [7]. Moreover, this study sample was restricted to hospitalized AMI cases and used the time of hospital admission instead of onset of AMI symptoms, which may have influenced the results. Potential meteorological confounders such as air temperature, relative humidity, and barometric pressure were not analyzed.

A recent study from Croatia on 2,412 hospitalized AMI survivors confirmed the significant increase of AMI incidence for the first 4 workdays after spring transition with a particular excess on Monday [8]. In contrast to the Swedish results, the authors reported a significant increase after the autumn transition for the first four workdays with a peak on Tuesday and Thursday. However, this study did not include fatal AMI cases and consider meteorological variables as potential confounders.

A smaller study performed by Jiddou et al. [9] on 935 hospitalized U.S. AMI survivors finally found a significantly increased AMI incidence for the first day (Sunday) after spring transition but no significant effects in terms of the autumn shift. Limitations of this study refer to its small sample size, an exclusion of fatal AMI cases, the use of the time of hospital admission as AMI onset, and the lacking consideration of meteorological confounders.

In addition, it remains unclear to what extent findings can be generalized across countries with different geographical location, since latitude and longitude influence light–dark cycles and circadian rhythms of humans. Thus, the objective of this study was to examine the association of DST transitions with AMI incidence using data of coronary deaths and non-fatal AMIs recorded in the MONICA/KORA Myocardial Infarction Registry, located in Southern Germany.

## Methods

### Sample

In the present analysis, all cases (*n* = 25,499) of coronary deaths and non-fatal AMI aged 25–74 years recorded in the MONICA/KORA Myocardial Infarction Registry between 1 January 1985 and 31 October 2010 were included. The population-based myocardial infarction registry was implemented in 1984 as part of the WHO-MONICA (Monitoring Trends and Determinants in Cardiovascular Disease) project. After the termination of MONICA in 1995, the registry became part of the framework of KORA (Cooperative Health Research in the Region of Augsburg). Since 1984, all cases of coronary deaths and non-fatal AMI of the 25–74 year old inhabitants in the city of Augsburg and the two adjacent counties (about 600,000 inhabitants) have been registered. Data sources for hospitalized patients include 8 hospitals within the study region and 2 hospitals in the adjacent areas. Approximately 80 % of all AMI cases of the study region are treated in the study region’s major hospital, Klinikum Augsburg, a tertiary care centre offering invasive and interventional cardiovascular procedures, as well as heart surgery facilities. Methods of case finding, diagnostic classification of events, and data quality control have been described in more detail elsewhere [10–12]. In brief data on pre-hospital coronary deaths is based on the death certificates collected by the three public health departments within the study region. For patients who are hospitalized with an AMI, a comprehensive set of data on demographics, cardiovascular risk factors, medical history and AMI treatment is being collected by individual interview and chart review.

The study was approved by the ethics committee of the Bavarian Medical Association. All participants with non-fatal AMI submitted written informed consent before being enrolled in the study.

### Data collection

AMI survivors were interviewed by trained study nurses during their hospital stays after they have been transferred from the intensive care unit using a standardized questionnaire.

Patients were asked whether they are currently employed (yes/no), have ever smoked or have stopped smoking (current smoker/ex-smoker/never smoked), whether they were diagnosed as having high blood pressure, blood lipids or diabetes prior to the AMI event, and whether they had an AMI before. Self-reported history of hypertension, hyperlipidaemia, diabetes or recurrent AMI (yes/no) was only considered if the chart review confirmed these diseases. Body mass index (BMI) was determined by assessment of height and weight during the hospital stay. Obesity (yes/no) was defined as BMI > 30 kg/m^2^. Data on medication prior to AMI (antiplatelets, beta-blockers, calcium antagonists, angiotensin-converting enzyme inhibitors, lipid-lowering agents) were collected both from individual patient interviews and chart reviews in AMI survivors. Patients were asked for the exact date and time of symptom onset. This information was validated against the information from the medical chart.

For coronary deaths, information on re-infarction, medication prior to AMI, current occupation, history of hypertension, hyperlipidemia, diabetes, smoking and obesity were requested from the last attending physician. Date and time of hospital admission or death was used as equivalent for date and time of symptom onset.

In addition, air temperature, relative humidity, and barometric pressure were measured on an hourly basis by the Bavarian Air Monitoring Network at one background air monitoring site 7 km south of the Augsburg city center [13]. 24-h mean values were calculated if at least 75 % of the hourly values were available.

### Data analysis

#### Time series model

We used a time series approach to model the association between DST transitions and incidence of non-fatal AMI and coronary deaths. In specific, we applied generalized additive Quasi-Poisson models to accommodate a Poisson distribution with overdispersion for the daily cases of AMI. To assess the influence of DST transition, we included two indicator variables for the week after the spring and the autumn transition. Alternatively, we included indicator variables for the 3 days after the time shifts. As potential confounders, we considered a global time trend, temperature, relative humidity, barometric pressure, and indicators for month of the year, weekday and holidays. Model selection was based on a reduction of the generalized cross-validation criteria (GCV) and the absolute value of the sum of the partial autocorrelation function [14]. For the meteorological factors, we compared the levels of same day, up to four previous days and the average of these five days and chose the term which minimized GCV most. To model nonlinear confounder effects, we used penalized regression splines to optimize the degree of smoothness. The optimal degree was then kept fix to allow a better comparability with sensitivity models. The final model included the following covariates: time trend and previous two day mean relative humidity as regression splines with four and two degrees of freedom, respectively, previous two day mean temperature as a linear term and day of the week as categorical variable.

#### Excess model

We reduced the time series to the months around the time transition (March and April for the spring shift and September to November for the autumn shift) to construct a prediction model in order to assess the potentially higher rates caused by the time shifts. On that account, we reran the confounder selection for spring and autumn months separately excluding the data of the week following the transition. The optimized spring model included time trend and same day mean relative humidity as regression splines with six and three degrees of freedom, same day mean temperature as a linear term, and month and weekday as categorical variables. The optimized autumn model included time trend and same day mean temperature as linear terms, same day mean relative humidity as regression spline with three degrees of freedom, and month and weekday as categorical variables. We then applied the two regression models to the datasets with weeks following the spring and autumn transitions, respectively, to predict the expected numbers of AMI per day. The incidence rate ratio was assessed as observed over expected events per day and the mean per weekday and corresponding 95 % confidence intervals were calculated.

#### Effect modification

We conducted stratified analyses of the data sets reduced to spring and autumn months on the basis of the therefore optimized confounder models. In specific, we assessed effect modification by sex, age (≤65 vs. >65 years), working vs. not working, first vs. recurrent AMI, non-fatal vs. fatal AMI, history of diabetes, hypertension, hyperlipidemia, BMI (<=30 kg/m^2^ vs. >30 kg/m^2^), smoking status, and intake of the following medications: antiplatelets, beta-blockers, calcium antagonists, ACE inhibitors, and lipid lowering drugs. Since it was not possible to obtain the information on a number of these variables for all patients (e.g. employment status was only requested in the interview and therefore not available for people who died before being interviewed), most of the stratified analyses are restricted to a limited sample size ranging between 89.9 % (diabetes) and 53.3 % (smoking) of the total population.

#### Sensitivity analysis

To check the robustness of our time series results we conducted several sensitivity analyses. First, we replaced the continuous time trend variable by a fixed effect for the year (S1). Second, we considered the inclusion of a penalized spline for time trend implying an optimization of the degree of smoothness by the model (S2). Third, we additionally modeled previous two day mean temperature and relative humidity by penalized splines (S3). Fourth, we additionally incorporated indicator variables for months and holidays (S4). All statistical analyses were performed with R software, version 3.0.0, package “mgcv”.

## Results

The study sample consisted of 25,499 persons with coronary death or non-fatal AMI with a mean age of 62 years. One third of them died out of hospital, 20 % died within 24 h and 28 days and the remaining 50 % survived at least 28 days. Further sample characteristics are detailed in Table 1.

**Table 1:** Demographic and clinical characteristics of the sample (n = 25,499)

Variable	n	%
Sex		
Female	6,975	27
Male	18,524	73
Age [years] (mean ± std)		62.6 ± 9.2
≤65 years	13,522	53
>65 years	11,977	47
Survival		
Out-of-hospital deaths	7,637	30
Died within 24 h	3,954	16
Died within 24 h to 28 days	1,157	4
Survivors (>28 days)	12,751	50
Recurrent myocardial infarction^a^		
yes	5,527	24
no	17,401	76
Current occupation^a^		
yes	5,496	27
no	15,067	73
Smoking^a^		
Current smoker	5,157	38
Former smoker	3,857	28
Never-smoker	4,569	34
Body Mass Index >30 kg/m^2^		
yes	15,321	60
no	10,178	40
Hypertension^a^		
yes	14,998	66
no	7,846	34
Hyperlipidaemia^a^		
yes	12,667	57
no	9,692	43
Diabetes^a^		
yes	7,364	32
no	15,520	68
Antiplatelets^a^		
yes	5,798	28
no	14,877	72
Beta-blockers^a^		
yes	5,745	28
no	14,930	72
Calcium antagonists^a^		
yes	5,086	25
no	15,583	75
Angiotensin-converting enzyme (ACE) inhibitors^a^		
yes	3,992	22
no	14,223	78
Lipid-lowering agents^a^		
yes	3,052	17
no	15,152	83

^a^Percentages refer to the number of non-missing values. Percent missing information per variable ranges from 10.1 % (recurrent infarction) to 46.7 % (smoking)

Risk ratios (RR) and corresponding 95 % confidence intervals (CI) of the 3 day indicators and the week indicators after time shift on the daily numbers of AMI are presented in Table 2 for the whole year time series, the periods reduced to months around the time shifts as well as the sensitivity analyses. Unadjusted models indicated an increased AMI risk after the spring shift, especially for the 1 week indicator, but almost no association with autumn shift indicators. Adjustment for potential confounders resulted in slightly reduced effect estimates of spring shift indicators for the whole year time series, but did not affect indicator estimates of the reduced time series. We observed the strongest association with a RR of 1.102 (95 % CI 0.972–1.250) for the 3 days after the spring shift for the reduced time series and the confounder model optimized for the excess model. In general, the results were robust to alternative model specifications. The excess model indicated higher incidence rate ratios for the weekdays lower ratios for the weekend (Table 3). Monday showed higher incidence rate ratios after the spring transition, but lower after the autumn transition.

**Table 2:** Risk ratios (RR) and 95 % confidence intervals (CI) for incidence of acute myocardial infarction on the first 3 days and the first week after clock shift

	Spring transition				Autumn transition			
	3 days		1 week		3 days		1 week	
Model description	RR	[95 % CIs]	RR	[95 % CIs]	RR	[95 % CIs]	RR	[95 % CIs]
Unadjusted model, whole year	1.103	[0.979;1.243]	1.110	[1.014;1.215]	0.940	[0.826;1.069]	0.996	[0.906;1.096]
Unadjusted model, reduced to months around time shift^a^	1.065	[0.946;1.199]	1.074	[0.980;1.178]	0.969	[0.850;1.104]	1.030	[0.934;1.136]
Main confounder model, whole year^b^	1.071	[0.948;1.209]	1.081	[0.985;1.185]	0.947	[0.833;1.078]	1.004	[0.913;1.105]
Main confounder model, reduced to months around time shift^a,b^	1.080	[0.954;1.222]	1.069	[0.973;1.174]	0.984	[0.862;1.124]	1.038	[0.94;1.145]
Optimized confounder model for months around time shift^c^	1.102	[0.972;1.250]	1.077	[0.981;1.182]	0.981	[0.858;1.121]	1.025	[0.928;1.133]
S1: Year as categorical variable instead of trend	1.072	[0.949;1.211]	1.082	[0.987;1.187]	0.947	[0.832;1.078]	1.004	[0.912;1.104]
S2: Trend as p-spline	1.070	[0.948;1.209]	1.08	[0.985;1.185]	0.947	[0.832;1.078]	1.004	[0.913;1.105]
S3: Trend and meteorology as p-spline	1.070	[0.947;1.209]	1.079	[0.984;1.184]	0.946	[0.831;1.076]	1.003	[0.912;1.103]
S4a: Trend and meteorology as p-spline, indicators for month and holiday	1.071	[0.943;1.216]	1.071	[0.972;1.181]	0.969	[0.849;1.106]	1.029	[0.931;1.136]
S4b: Trend and meteorology as p-spline, indicators for month and holiday, reduced to months around time shift^a^	1.097	[0.966;1.246]	1.072	[0.975;1.18]	0.968	[0.847;1.108]	1.019	[0.921;1.127]

^a^March and April for spring transition and September to November for autumn transition

^b^Model adjusted for time trend and previous two day mean relative humidity as regression splines with four and two degrees of freedom, respectively, previous two day mean temperature as a linear term and day of the week as categorical variables

^c^Spring model adjusted for time trend and same day mean relative humidity as regression splines with six and three degrees of freedom, same day mean temperature as a linear term, and month and weekday as categorical variables. Autumn model adjusted for time trend and same day mean temperature as linear terms, same day mean relative humidity as regression spline with three degrees of freedom, and month and weekday as categorical variables

**Table 3:** Mean incidence rate ratios (IRR) and 95 % confidence intervals for incidence of acute myocardial infarction for each weekday of the first week

	Spring predicition model^a^	Autumn predicition model^b^
Weekday	IRR [95 % CI]	IRR [95 % CI]
Sunday	0.937 [0.732; 1.141]	0.881 [0.667; 1.095]
Monday	1.207 [0.915; 1.500]	0.850 [0.572; 1.129]
Tuesday	1.185 [0.852; 1.519]	1.135 [0.861; 1.409]
Wednesday	1.075 [0.854; 1.297]	1.077 [0.832; 1.321]
Thursday	1.098 [0.809; 1.388]	1.133 [0.883; 1.383]
Friday	1.203 [0.950; 1.457]	1.244 [1.035; 1.453]
Saturday	0.850 [0.657; 1.043]	0.892 [0.657; 1.127]

^a^Spring model adjusted for time trend and same day mean relative humidity as regression splines with six and three degrees of freedom, same day

^b^Autumn model adjusted for time trend and same day mean temperature as linear terms, same day mean relative humidity as regression spline with three degrees of freedom, and month and weekday as categorical variables

Subgroup analysis for the time interval of 3 days and 1 week after the spring transition indicated a significantly increased risk to experience an AMI after the spring transition in persons who took ACE inhibitors prior to the AMI (3 days: RR 1.489, 95 % CI 1.151–1.927; 1 week: RR 1.297, 95 % CI 1.063–1.582) (Table 4). In addition, an increased risk for men was observed in the first 3 days after transition which was barely significant (RR 1.155, 95 % CI 1.000–1.334). After the clock shift in autumn, patients with a prior infarction had a significantly increased risk to have a re-infarction (3 days: RR 1.319, 95 % CI 1.029–1.691; 1 week: RR 1.270, 95 % CI1.048–1.539).

**Table 4:** Risk ratios (RR) and 95 % confidence intervals for incidence of acute myocardial infarction on the first 3 days and the first week after clock shift. Significant risk ratios are highlighted in bold-face letters

	Spring transition^a^				Autumn transition^b^			
	3 days		1 week		3 days		1 week	
Model description	RR	[95 % CIs]	RR	[95 % CIs]	RR	[95 % CIs]	RR	[95 % CIs]
All	1.102	(0.972;1.250)	1.077	(0.981;1.182)	0.981	(0.858;1.121)	1.025	(0.928;1.133)
Men	1.155	(1.000;1.334)	1.106	(0.993;1.232)	1.008	(0.860;1.181)	1.040	(0.924;1.171)
Women	0.957	(0.738;1.240)	1.001	(0.831;1.205)	0.914	(0.708;1.18)	0.986	(0.814;1.195)
Age ≤65 years	1.030	(0.853;1.245)	1.018	(0.886;1.170)	0.980	(0.813;1.183)	1.069	(0.933;1.226)
Age >65 years	1.184	(0.983;1.426)	1.145	(0.997;1.316)	0.980	(0.807;1.192)	0.977	(0.841;1.135)
Working	0.957	(0.712;1.286)	1.074	(0.873;1.322)	0.940	(0.705;1.253)	1.008	(0.816;1.246)
Not working	1.126	(0.953;1.330)	1.079	(0.952;1.224)	0.952	(0.793;1.143)	0.991	(0.863;1.137)
Recurrent AMI	1.049	(0.792;1.389)	0.994	(0.807;1.225)	1.319	(1.029;1.691)	1.270	(1.048;1.539)
First AMI	1.101	(0.941;1.287)	1.094	(0.974;1.228)	0.864	(0.726;1.030)	0.940	(0.826;1.068)
Non-fatal AMI	1.140	(0.950;1.369)	1.083	(0.944;1.242)	0.949	(0.778;1.159)	1.020	(0.88;1.181)
Fatal AMI	1.062	(0.878;1.283)	1.072	(0.933;1.233)	1.018	(0.840;1.233)	1.035	(0.894;1.197)
Diabetes mellitus yes	1.082	(0.850;1.377)	1.075	(0.900;1.284)	0.992	(0.772;1.273)	0.966	(0.797;1.171)
Diabetes mellitus no	1.040	(0.878;1.233)	1.039	(0.917;1.178)	0.944	(0.790;1.127)	1.026	(0.900;1.169)
Hypertension yes	1.067	(0.898;1.267)	1.062	(0.935;1.205)	0.942	(0.786;1.13)	0.972	(0.847;1.114)
Hypertension no	1.072	(0.848;1.356)	1.076	(0.905;1.279)	0.956	(0.751;1.217)	1.090	(0.917;1.296)
Hyperlipidemia yes	0.985	(0.812;1.194)	1.015	(0.881;1.168)	0.945	(0.774;1.154)	0.973	(0.838;1.130)
Hyperlipidemia no	1.175	(0.955;1.446)	1.113	(0.952;1.300)	0.950	(0.763;1.182)	1.034	(0.880;1.216)
Body Mass Index ≤30 kg/m2	1.147	(0.971;1.354)	1.12	(0.990;1.266)	1.005	(0.848;1.192)	1.011	(0.889;1.151)
Body Mass Index >30 kg/m2	1.007	(0.816;1.242)	1.016	(0.871;1.186)	0.932	(0.748;1.161)	1.049	(0.895;1.230)
Current smoker	0.841	(0.62;1.1420)	0.870	(0.696;1.089)	0.955	(0.715;1.276)	1.211	(0.993;1.477)
Former smoker	1.185	(0.902;1.556)	1.143	(0.932;1.403)	0.966	(0.724;1.288)	0.950	(0.761;1.186)
Never-smoker	1.071	(0.797;1.440)	1.106	(0.893;1.369)	1.009	(0.749;1.359)	1.080	(0.869;1.342)
Antiplatelets yes	1.204	(0.932;1.554)	1.140	(0.939;1.385)	0.906	(0.688;1.192)	0.932	(0.756;1.148)
Antiplatelets no	1.047	(0.881;1.245)	1.048	(0.923;1.190)	0.973	(0.810;1.168)	1.023	(0.892;1.173)
Betablockers yes	1.021	(0.773;1.347)	1.026	(0.839;1.255)	1.009	(0.774;1.315)	0.961	(0.780;1.185)
Betablockers no	1.119	(0.945;1.324)	1.095	(0.966;1.242)	0.935	(0.779;1.122)	1.012	(0.885;1.157)
Calcium antagonists yes	0.995	(0.742;1.335)	1.077	(0.873;1.329)	0.922	(0.687;1.238)	1.000	(0.806;1.241)
Calcium antagonists no	1.125	(0.95;1.331)	1.076	(0.948;1.220)	0.967	(0.811;1.153)	0.996	(0.871;1.138)
ACE inhibitors yes	1.489	(1.151;1.927)	1.297	(1.063;1.582)	0.781	(0.562;1.086)	0.928	(0.732;1.177)
ACE inhibitors no	1.036	(0.865;1.241)	1.059	(0.928;1.208)	1.010	(0.839;1.214)	1.015	(0.882;1.168)
Lipid lowering drugs yes	1.151	(0.839;1.580)	1.163	(0.921;1.469)	0.761	(0.539;1.075)	0.784	(0.601;1.023)
Lipid lowering drugs no	1.129	(0.954;1.336)	1.105	(0.975;1.251)	1.000	(0.833;1.199)	1.042	(0.909;1.193)

^a^Spring model adjusted for time trend and same day mean relative humidity as regression splines with six and three degrees of freedom, same day

^b^Autumn model adjusted for time trend and same day mean temperature as linear terms, same day mean relative humidity as regression spline with three degrees of freedom, and month and weekday as categorical variables

*AMI* Acute myocardial infarction; *ACE* Angiotensin-converting enzyme

## Discussion

Our study is the first one which investigated the association of DST shifts and AMI incidence in Germany and considered meteorological variables as potential confounders. In contrast to previous studies, we found no significant changes of AMI incidence in the entire sample for the first 3 days and 1 week after the spring transition to DST and the autumn transition from DST [6–9]. Analyses for each weekday separately yielded increased risks for Mondays and Tuesdays after the spring transition and reduced risks for Mondays after the autumn transition which are consistent with the Swedish results [6]. Although risk ratios in our study were greater compared with the estimates reported by Janszky et al. [6], they are lacking statistical significance due to smaller sample size.

Investigators of previous studies commonly explained the adverse associations of transitions to and from DST with AMI incidence by the disruption of the human circadian system, which may be associated with sleep deprivation particularly in the spring transition. Sleep deprivation itself negatively affects the cardiovascular system by increase of the sympathetic activity and proinflammatory biomarkers [15, 16]. The ability to adapt to DST may also depend on the chronotype. Chronotype is an attribute reflecting at what time of the day a persons’ physical functions, including sleeping, are active, change or reach a certain level. People can be divided into early (morning) and late (evening) chronotypes. Some studies reported that late chronotypes have more problems in adjusting to the spring transition to DST [17, 18], whereas the fall transition was more disturbing for early chronotypes [18]. In contrast, a recently published study found no differential adjustment of the chronotypes to DST transitions [19]. Chronotypes have been shown to depend not only on genetic factors and age [20, 21]. Studies indicated that the distribution of chronotypes among a population varies according to geographical location [22]. Latitude and longitude are key factors that influence how light–dark cycle interacts with the circadian rhythm of humans. It seems possible, that the different geographic locations of the studies which investigated the association of AMI incidence and DST shifts, namely Sweden, USA, and Croatia, may contribute to the difference of the results compared with our data from South Germany. To give an example, Sweden lies between latitudes 55° and 70°N whereas Augsburg is located at the 48°N latitude. On spring DST transition in 2010, sunrise was 30 min earlier in Stockholm than in Augsburg. Thus, individuals with late chronotypes in Sweden may experience a greater reduction in sleep duration than in Augsburg, leading to an excess of AMI cases.

Although no overall significant effects were found in our study, subgroup analyses revealed a significantly higher AMI incidence 3 days after the spring transition for persons with ACE inhibitor medication prior to infarction. Notably, the 1.489-fold increased risk was the highest estimate among all subgroup analyses. Janszky et al. [7] also found an increased risk for persons with prior ACE inhibitors (incidence ratio 1.141) but this result failed to be statistically significant. However, in contrast to Janszky et al. [7], in our study beta-blockers and calcium channel blockers tended to be associated with a lower risk for the spring transition. Thus, our findings do not fully support the hypothesis that prior cardiac medication may reflect more-progressed coronary atherosclerosis which increases the persons’ vulnerability to external AMI triggers such as time shifts.

In contrast to Janszky et al. [6, 7] but in line with Culic [8] we found men to be more prone to the spring shift. This may be explained by the fact that men are more likely to be later chronotypes than women also in later adulthood [21, 23]. The reasons for the conflicting study results are unclear. One may speculate that differences in study designs have contributed to these findings. In addition, as stated above, geographical location may be an overall factor that affects the association of DST transitions and AMI incidence.

In terms of the autumn DST transition, we found that patients with previous infarction had a higher risk to have a re-infarction in the week following autumn shift from DST compared with patients without prior AMI. This finding may also be related with the more-progressed coronary atherosclerosis and the possibility that minor disruptions of the circadian system, even if they may be associated with a longer sleep duration rather than a sleep deprivation, serve as a trigger of an AMI event.

The strengths of this study include the population-based sample of consecutive cases with coronary death and survivors with validated AMI, inclusion of patients in a defined area and according to defined criteria, and the standardized collection of risk factors, treatments and mortality data. Contrary to most prior investigations which were restricted to hospitalized patients with AMI [7–9], we have also considered coronary deaths and used the time of symptom onset instead of time of hospital admission as indicator of AMI onset [7, 9]. A major strength is the adjustment of the regression models for air temperature, relative humidity, and barometric pressure, which were reported to trigger AMI events [13]. Moreover, in the subgroup of AMI survivors, we were able to consider a number of characteristics which might influence the effect of DST on AMI incidence.

Our study shares one major limitation with previous studies on the same subject, namely the lack of data regarding sleep duration and quality, and chronotype. Besides weather data, no other potential triggers such as physical exertion, emotional states, or distress could be considered. Most of the subgroup analyses could only be performed for persons who were hospitalized and lived long enough to be interviewed. Thus, the results of the subgroup analyses can only be applied to AMI survivors. The statistical power of those analyses was limited and we cannot exclude that the significant findings may also be the result of type I error associated with multiple testing. In addition, the excess model for single weekdays has limited statistical power. Finally, the study does not include patients older than 74 years.

## Conclusions

In conclusion, our study showed no significant overall change of AMI risk associated with shifts to and from DST among the population in Southern Germany. However, the results indicate that specific subgroups such as men and persons with a history of AMI or prior treatment with ACE inhibitors may have a higher risk for AMI during DST shift. Further studies which include data on chronotype and sleep duration are needed in order to confirm these results.
